# Exome Sequencing and Genetic Testing for MODY

**DOI:** 10.1371/journal.pone.0038050

**Published:** 2012-05-25

**Authors:** Stefan Johansson, Henrik Irgens, Kishan K. Chudasama, Janne Molnes, Jan Aerts, Francisco S. Roque, Inge Jonassen, Shawn Levy, Kari Lima, Per M. Knappskog, Graeme I. Bell, Anders Molven, Pål R. Njølstad

**Affiliations:** 1 Department of Clinical Medicine, University of Bergen, Bergen, Norway; 2 Center for Medical Genetics and Molecular Medicine, Haukeland University Hospital, Bergen, Norway; 3 Department of Biomedicine, University of Bergen, Bergen, Norway; 4 Department of Pediatrics, Haukeland University Hospital, Bergen, Norway; 5 Faculty of Engineering – ESAT/SCD, Leuven University, Leuven, Belgium; 6 Wellcome Trust Sanger Institute, Cambridge, United Kingdom; 7 Computational Biology Unit, Uni Computing, Uni Research, Bergen, Norway; 8 Department of Informatics, University of Bergen, Bergen, Norway; 9 HudsonAlpha Institute for Biotechnology, Huntsville, Alabama, United States of America; 10 Division of Medicine, Department of Endocrinology, Departments of Medicine and Human Genetics, Akershus University Hospital, Lørenskog, Norway; 11 Departments of Medicine and Human Genetics, The University of Chicago, Chicago, Illinois, United States of America; 12 Gade Institute, University of Bergen, Bergen, Norway; 13 Department of Pathology, Haukeland University Hospital, Bergen, Norway; National Cancer Institute, National Institutes of Health, United States of America

## Abstract

**Context:**

Genetic testing for monogenic diabetes is important for patient care. Given the extensive genetic and clinical heterogeneity of diabetes, exome sequencing might provide additional diagnostic potential when standard Sanger sequencing-based diagnostics is inconclusive.

**Objective:**

The aim of the study was to examine the performance of exome sequencing for a molecular diagnosis of MODY in patients who have undergone conventional diagnostic sequencing of candidate genes with negative results.

**Research Design and Methods:**

We performed exome enrichment followed by high-throughput sequencing in nine patients with suspected MODY. They were Sanger sequencing-negative for mutations in the *HNF1A, HNF4A, GCK, HNF1B* and *INS* genes. We excluded common, non-coding and synonymous gene variants, and performed in-depth analysis on filtered sequence variants in a pre-defined set of 111 genes implicated in glucose metabolism.

**Results:**

On average, we obtained 45 X median coverage of the entire targeted exome and found 199 rare coding variants per individual. We identified 0–4 rare non-synonymous and nonsense variants per individual in our *a priori* list of 111 candidate genes. Three of the variants were considered pathogenic (in *ABCC8*, *HNF4A* and *PPARG,* respectively), thus exome sequencing led to a genetic diagnosis in at least three of the nine patients. Approximately 91% of known heterozygous SNPs in the target exomes were detected, but we also found low coverage in some key diabetes genes using our current exome sequencing approach. Novel variants in the genes *ARAP1*, *GLIS3*, *MADD*, *NOTCH2* and *WFS1* need further investigation to reveal their possible role in diabetes.

**Conclusion:**

Our results demonstrate that exome sequencing can improve molecular diagnostics of MODY when used as a complement to Sanger sequencing. However, improvements will be needed, especially concerning coverage, before the full potential of exome sequencing can be realized.

## Introduction

MODY (maturity-onset diabetes of the young) is a heterogeneous group of diabetes caused by single gene defects in at least ten genes affecting pancreas development and beta-cell function [1], [2], [3]. The most common MODY forms are caused by mutations in the glucokinase gene (*GCK*) [4] and the hepatocyte transcription factor genes *HNF1A* and *HNF4A* [5], [6]. *GCK*-MODY (MODY2) is a mild disease manifesting as slightly elevated fasting glucose, well controlled without medical treatment, and no risk for late diabetes-associated complications [7], [8]. In contrast, *HNF1A*- and *HNF4A*-MODY (MODY3 and MODY1, respectively) typically lead to progressive beta-cell dysfunction and high risk for late complications and patients often benefit from sulfonylurea treatment [9], [10], [11]. *HNF1B*-mutations result in a syndromic diabetes form (MODY5), which includes renal failure, genital and pancreatic malformations, and liver dysfunction [12], [13] According to the OMIM database, mutations in seven other genes (*BLK, CEL, INS, KLF11, NEUROD1, PAX4, PDX1*) can cause inherited diabetes with a MODY phenotype. There are also other forms of monogenic diabetes such as neonatal diabetes that presents before six months of age and syndromic diabetes, in which other features than diabetes dominates the clinical pictures (reviewed in [1]).

Genetic testing in monogenic diabetes is important for diagnosis and treatment [1], [2], [3]. When MODY is suspected, the current approach involves PCR amplification and Sanger sequencing of candidate genes, frequently with an iterative approach based on clinical features. For example, most laboratories will first screen *HNF1A,* followed by *HNF4A* and *GCK* in subjects exhibiting the classical features of MODY; and first *GCK*, then *HNF1A* and *HNF4A*, if the diabetic phenotype is mild and fasting glucose 5.5–8.5 mmol/l [1], [2]. If the patient presents with renal dysfunction, urogenital or pancreatic malformations, *HNF1B* is usually the first gene that is tested [1], [2].

Although systematic studies are lacking, our experience is that molecular genetic testing reveals a mutation in one of the common MODY genes in about 50% of probands referred to our laboratory. The remaining cases would also benefit from a genetic diagnosis, but the cost of sequencing other candidate genes often precludes further testing. A standard, complete investigation of *HNF1A*, *HNF4A* and *GCK* includes sequencing of 31 exons, where each sequencing reaction must be evaluated separately. Hence, the current approach is expensive and time-consuming, and establishes a molecular diagnosis only among a limited number of genes. Whole-exome capture and high-throughput sequencing has a great potential to detect causal gene variants in dominant and recessive disorders as well as in diseases due to *de novo* mutations [14], [15], [16], [17], [18]. Here, we describe our experience using exome sequencing in MODY patients referred to us for genetic testing.

## Materials and Methods

### Ethics Statement

The study was approved by the Regional Ethical Committee for Medical Research West and performed according to the Helsinki Declaration. We obtained verbal and written informed consent from the study participants.

### Study Population

**Figure 1:**
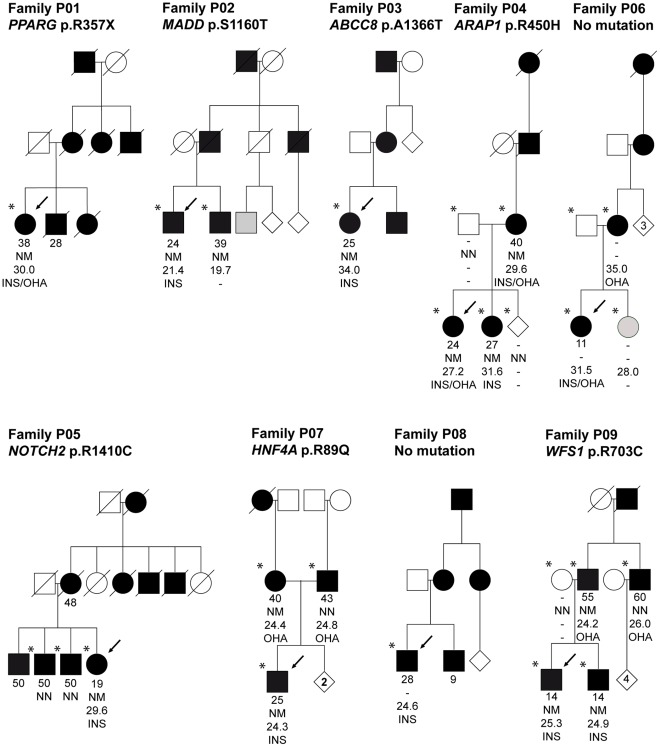
Partial pedigrees of the nine MODY families investigated. Squares represent male family members, circles female, and diamonds sex unknown. Numbers inside diamonds show the number of siblings. Black and grey symbols represent persons with diabetes and impaired glucose tolerance, respectively. An arrow denotes the proband in each family and stars indicate those subjects for whom DNA was available. Data under the symbols represent from top to bottom: age at diabetes diagnosis, mutation carrier status (N = Normal allele, M = Mutation), BMI and treatment (INS =  Insulin, OHA = Oral Hypoglycemic Agents).

We carried out whole-exome sequencing on nine probands with MODY of unknown cause recruited from the Norwegian MODY Registry (Figure 1 and Table 1). There was diabetes running in the families for at least three generations, autosomal dominant inheritance and age at diagnosis 11–28 years for at least one family member. All probands were negative for mutations in *HNF1A*, *HNF4A*, *GCK*, *HNF1B* and *INS* by Sanger sequencing.

**Table 1:** Clinical characteristics of the nine MODY probands investigated.

Proband	P01^a^	P02	P03	P04	P05	P06	P07	P08	P09
Sex	F	M	F	F	F	F	M	M	M
Age of diabetes diagnosis (y)	38^b^	24	25	24	19	11	25	28	14
Number of generations with DM	3	3	3	4	3	4	3	3	3
Current status									
Age (y)	68	52	38	31	55	16	35	35	29
BMI	30.1	21.4	29.9	27.2	28.3	31.5	24.3	24.6	25.3
Insulin dose (U/kg/day)	2.2	0.4	0	0.8	0.7	2.0	0.8	NA	0.5
OHA	Yes	No	Yes	Yes	No	Yes	No	No	No
Glycosylated hemoglobin (%)	7.5	6.1	9.0	6.7	7.1	8.4	7.2	9.3	6.4
Other clinical features									
Hypertension	Yes	No	No	No	Yes	Yes	Yes	No	No
Hypercholesterolemia	Yes	No	No	No	Yes	Yes	No	No	No
Retinopathy	Yes	No	No	No	Yes	No	Yes	No	No
Nephropathy	Yes	No	No	No	No	No	No	No	No
Arteriosclerosis	Yes	No	No	No	No	No	No	No	No
Polyneuropathy	Yes	No	No	No	Yes	No	No	No	No

None of the individuals had hearing loss or acantosis nigricans. Data regarding hepatic steatosis, renal cysts, polycystic ovarian disease and triglyceride status were not available in all individuals.

a P01 also had partial lipodystrophy with reduced subcutaneous fat in extremities and excess of abdominal subcutaneous fat. There was no evidence of hypertriglyceridemia, hepatic steatosis, acanthosis nigricans or polycystic ovarian disease.

b An affected family member was diagnosed at age 28 years.

Abbreviations: BMI, body mass index; DM, diabetes mellitus; NA, not available; OHA, Oral Hypoglycemic agents; U, international unit (used for insulin doses); y, year; kg, kilo gram body weight.

### Targeted Capture and Massive Parallel Sequencing

Targeted capture and massive parallel sequencing were performed at HudsonAlpha Institute for Biotechnology (Huntsville, AL) (File S1). In brief, SureSelect Human All Exon Kit (Agilent Technologies**,** Santa Clara, CA) was used for exome enrichment, and sequencing was performed on Genome Analyzer GAIIx (Illumina Inc., San Diego, CA). Samples were sequenced on one lane for paired-end 72-bp reads, and the samples with lowest yield were complemented with one additional lane of single-read 76-bp reads (P01, P03, P04, P05 P07).

### Read Mapping and Variant Analysis

We mapped paired-end-reads and single-reads to the reference human genomes (UCSC NCBI37/hg19) using Burrows-Wheeler Alignment tool (BWA) [19] (see also supporting Materials and Methods File S1). PCR duplicates were removed with PICARD (http://picard.sourceforge.net) followed by base quality recalibration using GATK [20]. SNPs and indels were called by SAMtools [21] mpileup, correcting for overestimated mapping quality from BWA. SNPs were filtered by the following criteria: (1) SNPs should not be in a cluster with window-size of 10 bp, (2) sequencing depth should be at least 8 X; and (3) quality score should be ≥30. We used Annovar [22] (Nov 22, 2011) and in-house scripts to annotate and filter variants after variant calling. The performance of our exome sequencing variant calling pipeline was tested against heterozygous genotypes present in the enrichment target regions derived from the Affymetrix 6.0 whole-genome genotyping array in seven of the individuals (File S1).

### *In silico* Analyses of Candidate Variants

We evaluated possible functional significance of the variants using PolyPhen v2.0.23 [23], Align-GVGD (http://agvgd.iarc.fr) and SIFT (http://sift.jcvi.org/), see File S1.

### Selection of genes of Interest

We selected genes previously implicated in monogenic diabetes and related syndromes [1], [24], genes with important roles in the beta cell [25], [26] and genes implicated from whole-genome-significant SNP associations with type 2 diabetes (T2D) or fasting glucose [24], [27], [28], [29], [30], [31]. For the T2D and fasting glucose-associated regions, we selected named gene/genes highlighted in the respective publications. Notably, experimental evidence directly supporting that these genes are responsible for the associations, is mostly lacking. The 111 genes, of which 109 were included in the capture assay, totaled 272 kb of exonic sequence (Table S1).

### Rare Variant Validation

All variants in the candidate mutation set were validated by PCR amplification of the variant-containing exon from the original patient sample, followed by Sanger sequencing. Frequency estimates were generated by genotyping 340 Norwegian healthy controls using the MassARRAY iPLEX system or by Sanger sequencing.

## Results

By sequencing the exomes of the nine MODY probands (Table 1, Figure 1), we obtained 3.5–5.8 Gb mapable sequence per sample with 36–57 X median coverage of the targeted exome and 88–93% of the exome targeted at least eight times (Table 2). The candidate diabetes genes showed similar coverage (Tables S1 and S2). The exceptions were *DGKB* and *THADA*, which were not present on the exome enrichment array highlighting one problem with our approach: current target capture reagents may not include all exons of interest. The MODY genes *HNF4A* and *HNF1B* showed relatively good coverage (≥87%, at 8X) throughout the entire coding regions, while *GCK* (83%), *HNF1A* (72%) and *INS* (58%) were less uniformly covered (Tables S1 and S2).

**Table 2:** Overall exome coverage and target gene set coverage statistics.

			Percentage at ≥			Percentage at ≥	
Sample ID	Unique Gbs aligned	Median (X)	8X	20X	Median (X)	8X	20X
P01	3.5	36	89	71	33	85	66
P02	3.5	36	88	71	34	86	68
P03	4.8	50	92	79	46	89	75
P04	5.5	57	92	81	52	90	77
P05	5.4	55	92	8	49	89	76
P06	3.6	39	89	73	35	85	68
P07	5.8	59	93	82	55	90	78
P08	3.7	39	89	73	37	86	69
P09	3.5	37	89	71	35	86	68
Average	4.4	45	90	76	42	87	72

We identified an average of 14,463 substitutions and indels per sample (in the targeted exome) after quality control (Table 3). The quality of the data was investigated by comparisons from 7,800 genotyped SNPs, present in the Agilent capture region, and obtained from the Affymetrix 6.0 genotyping array for seven of the nine individuals. Between 89 and 92% of the heterozygous genotyping-array-SNPs present in the capture regions were detected (File S1 and Table S3).

**Table 3:** Overview of all substitutions and indels detected in the nine probands before and after variant reduction.

	Patients									
Variants and filter	P01	P02	P03	P04	P05	P06	P07	P08	P09	Average
All exonic	14102	14219	14773	14974	14668	14253	14720	14190	14269	14463
Exonic coding	6460	6582	6776	6855	6634	6526	6744	6557	6565	6633
Not in in-house database (50 samples)	250	215	263	255	252	255	264	267	260	253
Not in 1000 G >0.5%	193	183	197	202	197	197	202	213	206	199
Candidate variants in 111 target genes	1	2	3	1	2	0	1	0	4	2

Next, we developed a data reduction pipeline consisting of several steps (Table 3). We first excluded all variants not present in the actual coding sequence or in splice sites; and synonymous variants other than those occurring at canonical splice sites. We subsequently filtered against an in-house database of genetic variants from 50 Norwegian whole exomes and finally excluded variants with minor allele frequencies >0.5% of the 1000 Genomes Project. This reduced the number to 183–213 rare, coding single-nucleotide substitutions and coding indels per individual (Table 3). Combining all nine individuals, this resulted in 1,733 different variants located in 1,569 different genes. Only 50 variants were present in more than one of the nine individuals. On the gene level, 266 genes were listed with rare (most often different) variants in more than one individual, and 24 genes with rare variants in three or more individuals. Hence, despite our rigid procedure for variant filtration, a large number of potential candidate genes emerged from the nine patient data sets.

We then focused on the candidate gene list (Table S1). We identified 14 rare coding variants in 12 genes of the 111 candidate genes. Thirteen of the variants were verified by Sanger sequencing (Tables 3 and 4). Frequency estimates in 340 healthy controls, computational methods to estimate deleteriousness and literature searches were performed for the remaining 13 variants (Table 4). When available, additional family members were sequenced for variants not present in either the 1000 G database, dbSNP or in our 340 Norwegian controls (Figure 1).

**Table 4:** Rare coding variants identified in the 111 target candidate genes using whole exome sequencing in nine patients with suspected MODY.

Gene	Chr:Position	Variant	dbSNP132/1000 G^b^frequency	Frequency in340 Norwegiancontrols	SIFT/PolyPhen-2/AlignGVGD^a^	Patient	Conclusion
*ABCC8*	11∶17418486	c.4096G>A/p.A1366T	−/0	0	−/+/C55	P03	Pathogenic
*ALMS1*	2∶73677199	c.3542C>T/p.T1181I	−/0	0.1%	n.a/+/n.a.	P09	
*ARAP1*	11∶72421497	c.1349G>A/p.R450H	−/0	0	+/−/C0	P04	
*CRY2*	11∶45893711	c.1528G>C/p.G510R	−/0	0.1%	−/−/C15	P02	
*GLIS3*	9∶4286332	c.94C>G/p.R32G	−/0	0	+/+/C0	P03	
*HADH*	4∶108940732	c.456G>T/p.Q152H	rs1051519/0.2%	-	−/−/C0	P09	
*HNF4A*	20∶43034848	c.266G>A/p.R89Q	−/0	0	+/++/C35	P07	Pathogenic
*MADD*	11∶47317569	c.3479G>C/p.S1160T	−/0	0	−/++/C55	P02	
*NOTCH2*	1∶120468211	c.4228C>T/p.R1410C	−/0	0	+/+/C25	P05	
	1∶120478125	c.3625T>G/p.F1209V	−/0	0.4%	+/+/C45	P03	
	1∶120548095	c.272G>T/p.R91L	FALSE	FALSE	FALSE	P05	False positive
*PPARG*	3∶1258536	c.1071G>A/p.R357X	−/0	0	Nonsense	P01	Pathogenic
*SREBF1*	17∶17718592	c.2435G>A/p.R812Q	−/0	1.0%	−/−/C0	P09	
*WFS1*	4∶6354530	c.2107C>T/p.R703C	−/0	0	+/++/C65	P09	

a SIFT: − tolerated, + not tolerated/PolyPhen-2: − benign, + possibly damaging, ++ probably damaging/Align-GVGD: the Grantham variation (GV), and the Grantham deviation (GD) are combined to provide graded classifiers from most likely to interfere with function (class C65) to least likely (class C0).

b Allele frequencies from the interim analysis of phase I of the 1000 Genomes Project, 2010.08.04 sequence index, which included 629 samples (SNPs released in November 2010, indels released in February 2011).

Abbreviation: Chr, chromosome number; 1000 G, the 1000 Genomes Project; n.a, not analysed due to insufficient number of alignments to make prediction.

### Exome Sequencing Reveals Three Variants in genes Known to Cause Autosomal Dominant Disease

In subject P01, we identified the heterozygous nonsense mutation c.1071G>A/p.R357X introducing a premature stop codon in *PPARG* exon 7. This mutation has previously been shown to cause severe insulin-resistant diabetes and partial lipodystrophy [32], [33], [34]. The proband’s age at diagnosis was 38 years; she was included in our study because a family member was diagnosed at age 28 years. The proband’s BMI was 27.6 kg/m^2^ in 2002, 30.1 kg/m^2^ in 2010, and her insulin requirement has in the same period increased from 1.4 to 2.2 U/kg/day. There was a high prevalence of micro- and macro-vascular complications in most of the affected family members, although none were available for genetic testing (Table 1, Figure 1). Thus, at recruitment the proband had a MODY phenotype, but was insulin-resistant on follow-up. The same mutation has been reported in patients with a similar clinical picture [32], [33], [34]. We consider this mutation pathogenic.

In subject P03, we detected the novel and heterozygous non-synonymous *ABCC8* mutation c.4096G>A/p.A1366T. Amino acid 1366 is highly conserved and located in the ATP-binding domain. Other nearby amino acid substitutions are associated with either congenital hyperinsulinism or neonatal/adult-onset diabetes [35], [36]. The proband was diagnosed with diabetes at 25 years of age and is currently treated with sulfonylurea and metformin. All four diabetic family members (none available for genetic testing) were treated with OHA. The proband’s age at diagnosis was late for *ABCC8* diabetes although other such cases have been described [35], [37], [38], [39]. The large size of *ABCC8* makes it less amenable to Sanger-based mutation screening, which may underestimate the role of this gene in MODY. Since the proband was sulfonylurea-sensitive, we categorized p.A1366T as being probably pathogenic. Further sequencing studies of unselected MODY patients will elucidate if *ABCC8* is a more common cause of MODY than previously anticipated.

In subject P07, we identified a novel non-synonymous mutation c.266G>A/p.R89Q in *HNF4A*. This was surprising since *HNF4A* already had been screened. Sanger re-sequencing confirmed the mutation. When re-examining the first electropherogram, the mutation was detectable. Hence, it had been overlooked. Another substitution of the same codon, c.265C>T/p.R89W, has been identified in MODY [40]. The amino acid residue at position 89 of HNF4A is highly conserved from *Drosophila* to humans and part of the DNA binding domain. Both parents are of normal weight but developed diabetes in their early forties (Figure 1). Sanger sequencing revealed that the mutation was inherited from the maternal side of the family that appears to have a stronger history of diabetes. After receiving the molecular diagnosis the proband made a successful transfer from insulin to sulfonylurea. We consider the mutation p.R89Q pathogenic.

### Other Rare Variants in the Candidate Gene Set

In the 111 candidate genes, we also identified novel (not present in 50 in-house exomes, 340 healthy controls or 1000 Genomes) variants in potentially interesting genes implicated in susceptibility to diabetes, albeit thus far not in an autosomal dominant mode of inheritance: *ARAP1*, *GLIS3*, *MADD*, *NOTCH2* and *WFS1* (Table 4). Each genetic variant is discussed in some detail below.

In subject P02, we detected the novel and heterozygous non-synonymous *MADD* variant c.3479G>C/p.S1160T. The MADD (MAP-kinase activating death domain) protein is known to have a role in apoptosis [41] and SNPs in the *MADD* region are associated with elevated pro-insulin and fasting glucose levels [42]. The subject was diagnosed with diabetes at 24 years of age. He has for ten years been treated with sulfonylurea and is currently on insulin (0.5 U/kg/day). C-peptide and proinsulin were detectable, however, not elevated. The affected and lean brother also carried the variant. He was diagnosed with diabetes 39 years old and is treated with oral hypoglycaemic agents (OHA). No other family members were available for genetic analysis.

Individual P03 (who had a probably pathogenic *ABCC8* mutation) also had a potentially interesting variant in *GLIS3* which is a transcription factor expressed in beta-cells and important for insulin gene expression [43]. Mutations in *GLIS3* can cause a recessive form of neonatal diabetes and congenital hypothyroidism (OMIM#610199) [44], [45]. There are, however, no reports on dominant *GLIS3* mutations, and the variant is located in a protein region with no known function. With only the proband available for genetic testing, it was not possible to study the segregation of the variant in the family. We consider that the *ABCC8* mutation is more likely to be the pathogenic variant in this patient.

In subject P04, we detected the *ARAP1* (previously *CENTD2*) variant c.1349G>A/p.R450H. It is predicted to be benign by PolyPhen and AlignGVGD (Table 4) but damaging by SIFT, and it co-segregates with diabetes in the core family (Figure 1). Common variants at this locus have been associated with type 2 diabetes, fasting glucose and pro-insulin level [28], [46], [47], and it is suggested that this effect is mediated through reduced insulin secretion capacity [46], [47]. However, as with most GWAS-associated regions, the causative variant has not yet been pinpointed, and the nearby *STARD10* gene was recently suggested as a better biological candidate gene in the region [47]. Although not on our original candidate gene list, in retrospect, no rare variants were detected in *STARD10*.

The *NOTCH2* variant c.4228C>T/p.R1410C, found in P05, did not co-segregate with diabetes (Figure 1). This gene was implicated as a type 2 diabetes locus in a recent GWAS meta-analysis [28]. It is also known that heterozygous mutations in *NOTCH2* can cause Alagille syndrome (OMIM#610205) and Hajdu-Cheney syndrome (OMIM #102500). The patient showed no symptoms suggesting any of these diseases.

In subject P09, we detected the novel *WFS1* variant c.2107C/T/p.R703C. The affected amino acid residue is strongly conserved and the variant is suggested to be probably damaging by all three prediction programs (Table 4). Recessive mutations in *WFS1* can lead to Wolfram syndrome (OMIM #222300), which includes diabetes, hearing impairment and psychiatric disease, while heterozygous carriers appear to show no major symptoms associated with diabetes. *Wfs1* null mice and genetic association studies suggest a role in insulin secretion [48], [49]. The proband developed diabetes 14 years old with no type 1 auto-antibodies and currently requires 0.5 U/kg/day insulin. There was no familial hearing impairment. The affected brother and father carried the variant, but not the affected uncle (Figure 1). Age-of-diagnosis and insulin requirements are distinctly different between the affected brothers and their father and uncle.

## Discussion

Exome sequencing has shown a great potential for identification of disease mutations in monogenic disorders [14], [15], [16], [17], [18]. However, it is not clear how representative the early proof-of-principle studies are and whether this technology is ready to replace or complement traditional Sanger sequencing for clinical genetic testing.

Here, we show that exome sequencing can provide a significant diagnostic advantage in a substantial fraction of patients where Sanger sequencing often is inefficient, such as cases with atypical clinical presentation (family P03) or when clinical information is limited (family P01). For this group of patients, exome sequencing is an attractive option compared to the current “phenotypically” driven genetic testing as it allows testing beyond the short list of genes typically tested by Sanger sequencing.

To illustrate how this technology could be utilized for routine diagnostic use, we restricted our analysis to a list of 111 candidate genes. Our list included known disease genes for monogenic diabetes, insulin resistance and diseases related to glucose homeostasis. We also explored rare variants in candidates such as genes encoding transcription factors important for pancreatic development and islet specification/differentiation [25], [26], and genes identified in GWAS of diabetes and fasting glucose levels. Our study identified some rare variants in the latter gene categories (Table 4 and results section). Although these variants are located in attractive candidates, to claim causality would obviously require much more extensive proof than for genes already known to cause autosomal-dominant diabetes. Such evidence would include co-segregation between variant and disease in large families, the presence in other subjects with a similar phenotype together with functional and clinical studies. Especially the limed number of available family members for segregation analysis, does not allow us to reach this level of support for the variants in our “candidate gene” category. Hence, we could not determine whether these variants are causing MODY or at least may act as polygenic risk factors that warrant further investigations (Table 4).

A possible advantage with exome sequencing is that it also allows for an extensive search for completely novel diabetes genes in individuals with no genetic defect in the known diabetes genes. However, for diabetes, where the genes for several monogenic forms already have been detected [1], the search for remaining, unmapped disease loci will be hampered by significant locus- and clinical heterogeneity. Our registry-based clinical sample with limited access to extended pedigrees was not powered to identify novel disease genes among the approximately 200 rare coding variants in each individual. Thus, international efforts to sequence the entire exomes of larger numbers of carefully selected subjects and to identify large multi-generational diabetes families may be a way forward.

For diagnostic utility, our study reveals that exome sequencing can increase the possibility for a genetic diagnosis in MODY. The coverage for certain key genes must, however, be improved before exome sequencing can replace Sanger sequencing in routine molecular diagnostics. Recent and ongoing improvements in capture hybridization and high-throughput sequencing technologies are promising, but the coverage problem may not be solved completely by new enrichment kits, higher read depths and longer reads. In the meantime, it might be attractive to use tailored hybridization capture for the disease of interest followed by very high-coverage sequencing of the disease-specific gene panels [50]. The increased coverage for the target genes must be weighted against the cost of developing, optimizing and keeping up-to-date disease-specific gene panels and the limited ability to detect unexpected phenotype-genotype correlations.

In conclusion, we consider phenotypically driven Sanger sequencing still as the first choice for genetic testing in patients with classical features of MODY. Exome sequencing is currently an important complement when Sanger sequencing is negative, or in patients with atypical clinical presentation. In the near future, we believe that tailored hybridization capture for selected genes of interest and very high-coverage sequencing of specific gene panels will replace Sanger sequencing. Ongoing refinements in the design of capture reagents, sequencing technologies and bioinformatics will, however, most likely ultimately lead to exome and possibly whole-genome sequencing as state-of-the art in molecular diagnostics of MODY.

## Supporting Information

File S1 **Supplementary Materials and Methods.**
(DOC)

Table S1 **Candidate genes with reason for inclusion and average coverage in the nine tested samples.**
(DOC)

Table S2 **Fraction of target bases covered at minimum 8 X for each sample and gene.**
(DOC)

Table S3 **Comparison between heterozygous genotypes obtained from the Affymetrix 6.0 chip and exome sequencing.**
(DOC)
