# Supplementary material for: Exome Sequencing and Genetic Testing for MODY
Source: PLoS One. 2012 May 25;7(5):e38050. doi: 10.1371/journal.pone.0038050 (PMC3360646; doi:10.1371/journal.pone.0038050)
Supplement: File S1 — Supplementary Materials and Methods. (DOC) [file pone.0038050.s001.doc]

**Supplementary Information File 1. Materials and Methods**

**Targeted capture and massive parallel sequencing**

Targeted capture and massive parallel sequencing were performed at the HudsonAlpha Institute for Biotechnology (Huntsville, AL). Approximately 180 000 exons were captured from 4 µg genomic DNA in nine patients, using the SureSelect Human All Exon Kit, according to the manufacturer’s protocol (Agilent Technologies**,** Santa Clara, CA). DNA was sheared by nebulization and fragmented DNA ends were repaired and universal adapters ligated onto the repaired ends (NEBNext DNA Sample Prep, New England Biolabs, Ipswich, MA). The DNA library was purified and fragments were subsequently amplified by PCR. The DNA library was captured by hybridization to the biotinylated RNA library baits. Hybridized genomic DNA was purified with Dynal (Invitrogen) streptavidin-coated magnetic beads and re-amplified. Exome sequencing of the DNA library was performed on Genome Analyzer GAIIx (Illumina Inc., San Diego, CA). Each sample was sequenced on one lane for paired-end 72-bp reads and five samples (P1, P3, P4, P5, P7) were also sequenced for one lane of single-end 76-bp reads.

**Read mapping and variant analysis**

We mapped paired-end sequencing reads to the reference human genomes (UCSC NCBI37/hg19) using Burrows-Wheeler Alignment tool (BWA) . In the resulting BAM files, we removed PCR duplicates using the PICARD tool (http://picard.sourceforge.net) followed by a base quality recalibration using GATK . In this step, we corrected for ReadGroup, QualityScore, Cycle and Dinucleotide with a maximum of 20,000 reads per locus. We called SNPs and indels using SAMtools mpileup with the –C50 option to correct for overestimated mapping quality from BWA. The variant list was annotated using GATK VariantAnnotator . Finally, SNPs were filtered using the following criteria: (1) SNPs should not be in a cluster with window-size of 10 bp, (2) depth should be at least 8X and (3) quality score should be more than 30. We used the Annovar software tools (version June 18, 2011) and in-house scripts to annotate and filter variants after variant calling.

The performance of our exome sequencing variant calling pipeline was tested against heterozygous genotypes present in the enrichment target regions derived from the Genome-Wide Human SNP Array 6.0 (Affymetrix 6.0). Seven out of the nine samples in the present study were genotyped using the Affymetrix 6.0 in a larger batch of 75 samples. High-quality SNPs calls were extracted using standard QC measures (>0.95 % SNP missingness, and minor allele frequency ≥0.05) yielding a total of 774068 good quality common SNPs. SNPs located in the exome enrichment target regions were extracted yielding a total of 7800 SNPs located in the target regions. To avoid possible strand issues, only heterozygous calls were extracted resulting in an average of 2228 high quality Affymetrix 6.0-derived heterozygous SNPs per individual (range 2143-2310 heterozygous calls per individual). Concordance rate and false negative rates were calculated by comparing the high-quality Affymetrix 6.0-derived heterozygous genotypes from the exome enrichment regions with the exome sequencing calls from the standard pipeline. An average of 90.6% of the heterozygous target region Affymetrix genotype calls were called also on the exome-sequencing pipe-line (99.95% concordant calls).

***In silico* analyses of candidate mutations**

We evaluated possible functional significance of the various mutations using Polymorphism Phenotyping v2.0.23 (PolyPhen-2) , Align-GVGD (http://agvgd.iarc.fr) and SIFT (http://sift.jcvi.org/). The PolyPhen-2 software classifies the functional effect of a missense mutation into three categories (probably damaging, possibly damaging and benign) by considering the evolutionary conservation and the biophysical nature (side-chain composition, polarity and molecular volume) of the amino acids involved, as well as the proximity of the substitution to important functional domains or structural sites of the protein. Align-GVGD (http://agvgd.iarc.fr) is an extension of the Grantham matrix, combining the amino acid biophysical characteristics and protein multiple sequence alignment (MSA). The output is in the form of two variables; the Grantham variation (GV), which scores the extent of variation (species conservation) between residues at a given position in the MSA, and the Grantham deviation (GD), a measure of the biochemical distance between this group of residues and the mutant amino acid. The GV and GD scores are combined to provide graded classifiers from most likely to interfere with function (class C65) to least likely (class C0). The MSAs were constructed with ClustalX v.2.0.12 and 3D-Coffee using protein sequences available through the HomoloGene database (NCBI). Alignments were composed of up to six mammalian species and to the depth of zebrafish or *Drosophila*.

**Data-reduction pipe-line**

We identified an average of 14,463 substitutions and indels per sample after quality control. We developed a data reduction pipeline consisting of several steps. First, we eliminated variants not present in the actual coding sequence or in splice sites and synonymous variants other than those occurring at canonical splice sites. Next we filtered against an in-house database of genetic variants from 50 other individuals (all non-diabetic) obtained from our other exome sequencing projects. We thereafter excluded all variants present at a minor allele frequency (MAF) >0.5 % in the 1000 Genomes phase 1 project data 2010.08.04 sequence index, which included 629 samples (26.1 million SNPs released in November 2010 and 3.7 million indels released in February 2011). This further reduced the number of rare mutations to an average of 199 rare single nucleotide substitutions and coding indels per individual.

1. Durbin RM, Abecasis GR, Altshuler DL, Auton A, Brooks LD, et al. (2010) A map of human genome variation from population-scale sequencing. Nature 467: 1061-1073.

2. McKenna A, Hanna M, Banks E, Sivachenko A, Cibulskis K, et al. (2010) The Genome Analysis Toolkit: a MapReduce framework for analyzing next-generation DNA sequencing data. Genome Res 20: 1297-1303.

3. Li H, Handsaker B, Wysoker A, Fennell T, Ruan J, et al. (2009) The Sequence Alignment/Map format and SAMtools. Bioinformatics 25: 2078-2079.

4. Wang K, Li M, Hakonarson H (2010) ANNOVAR: functional annotation of genetic variants from high-throughput sequencing data. Nucleic Acids Research 38: e164.

5. Adzhubei IA, Schmidt S, Peshkin L, Ramensky VE, Gerasimova A, et al. (2010) A method and server for predicting damaging missense mutations. Nat Methods 7: 248-249.

6. Larkin MA, Blackshields G, Brown NP, Chenna R, McGettigan PA, et al. (2007) Clustal W and Clustal X version 2.0. Bioinformatics 23: 2947-2948.

7. O'Sullivan O, Suhre K, Abergel C, Higgins DG, Notredame C (2004) 3DCoffee: Combining Protein Sequences and Structures within Multiple Sequence Alignments. Journal of Molecular Biology 340: 385-395.
