# Supplementary material for: Exome Sequencing and Genetic Testing for MODY
Source: PLoS One. 2012 May 25;7(5):e38050. doi: 10.1371/journal.pone.0038050 (PMC3360646; doi:10.1371/journal.pone.0038050)
Supplement: Table S1 — Candidate genes with reason for inclusion and average coverage in the nine tested samples. (DOC) [file pone.0038050.s002.doc]

**Table S1**

Candidate genes with reason for inclusion and average coverage in the nine tested samples

| Gene | Reason for  inclusion | Average amount of target at | | Average median  coverage |
| --- | --- | --- | --- | --- |
|  |  | ≥8X | 20X |  |
| *ABCC8* | NDM, CHI | 0.87 | 0.61 | 30 |
| *ADAM30* | GWAS | 1.00 | 1.00 | 83 |
| *ADAMTS9* | GWAS | 0.97 | 0.90 | 66 |
| *ADCY5* | GWAS | 0.86 | 0.62 | 28 |
| *ADRA2A* | GWAS | 0.62 | 0.50 | 22 |
| *AGPAT2* | Syndrome | 0.72 | 0.23 | 14 |
| *AKT2* | Syndrome | 0.90 | 0.57 | 25 |
| *ALMS1* | Syndrome | 0.98 | 0.94 | 79 |
| *APPL1* | Candidate | 0.92 | 0.81 | 55 |
| *ARAP1/CENTD2* | GWAS | 0.68 | 0.36 | 14 |
| *BCL11A* | GWAS | 0.93 | 0.73 | 36 |
| *BLK* | MODY | 0.90 | 0.65 | 29 |
| *BSCL2* | Syndrome | 0.96 | 0.84 | 39 |
| *C2CD4B* | GWAS | 0.05 | 0.00 | 2 |
| *CAMK1D* | GWAS | 0.97 | 0.85 | 55 |
| *CAV1* | Syndrome | 0.97 | 0.68 | 29 |
| *CDC123* | GWAS | 1.00 | 0.94 | 60 |
| *CDKAL1* | GWAS | 1.00 | 0.95 | 70 |
| *CDKN2A* | GWAS | 0.76 | 0.24 | 14 |
| *CDKN2B* | GWAS | 0.78 | 0.23 | 14 |
| *CEL* | MODY | 0.72 | 0.43 | 18 |
| *CHCHD9* | GWAS | 0.97 | 0.48 | 20 |
| *CISD2/WFS2* | Syndrome | 0.99 | 0.72 | 29 |
| *CRY2* | GWAS | 0.88 | 0.69 | 37 |
| *DGKB* | GWAS | Not present on the capture array | | |
| *DUSP8* | GWAS | 0.43 | 0.14 | 7 |
| *DUSP9* | GWAS | 0.58 | 0.46 | 19 |
| *EIF2AK3* | Syndrome | 0.99 | 0.93 | 75 |
| *FADS1* | GWAS | 0.84 | 0.73 | 46 |
| *FOXA1* | Candidate | 0.81 | 0.66 | 35 |
| *FOXA2* | Candidate | 0.64 | 0.44 | 19 |
| *FOXA3* | Candidate | 0.72 | 0.48 | 19 |
| *FTO* | GWAS | 0.94 | 0.90 | 71 |
| *FXN* | Syndrome | 0.75 | 0.72 | 41 |
| *G6PC2* | GWAS | 0.99 | 0.88 | 80 |
| *GATA4* | Candidate | 0.63 | 0.56 | 30 |
| *GATA6* | Candidate | 0.60 | 0.39 | 13 |
| *GCK* | MODY, NDM, CHI | 0.83 | 0.48 | 20 |
| *GCKR* | GWAS | 1.00 | 0.91 | 56 |
| *GIPR* | GWAS | 0.66 | 0.40 | 15 |
| *GLIS3* | NDM, GWAS | 0.91 | 0.79 | 46 |
| *GLUD1* | CHI | 0.94 | 0.89 | 82 |
| *HADH* | CHI | 0.91 | 0.77 | 43 |
| *HFE* | Syndrome | 0.98 | 0.72 | 53 |
| *HHEX* | GWAS | 0.60 | 0.44 | 16 |
| *HMGA2* | GWAS | 0.75 | 0.72 | 38 |
| *HNF1A* | MODY, GWAS | 0.72 | 0.39 | 16 |
| *HNF1B* | MODY, GWAS | 0.87 | 0.63 | 33 |
| *HNF4A* | MODY, CHI | 0.89 | 0.52 | 22 |
| *IDE* | GWAS | 0.96 | 0.88 | 55 |
| *IGF1* | GWAS | 0.99 | 0.85 | 40 |
| *IGF2BP2* | GWAS | 0.92 | 0.72 | 38 |
| *INS* | MODY, NDM | 0.58 | 0.10 | 10 |
| *INS/IGF2* | MODY, NDM | 0.48 | 0.12 | 8 |
| *INSM1* | Candidate | 0.43 | 0.15 | 8 |
| *INSR* | CHI | 0.91 | 0.79 | 48 |
| *IRS1* | GWAS | 0.86 | 0.51 | 21 |
| *ISL1* | Candidate | 0.96 | 0.79 | 41 |
| *ITGB6* | GWAS | 0.99 | 0.94 | 68 |
| *JAZF1* | GWAS | 0.91 | 0.74 | 52 |
| *KCNJ11* | NDM, CHI, GWAS | 0.99 | 0.81 | 32 |
| *KCNQ1* | GWAS | 0.71 | 0.46 | 18 |
| *KLF11* | MODY | 0.92 | 0.80 | 42 |
| *KLF14* | GWAS | 0.34 | 0.05 | 4 |
| *LGR5* | GWAS | 0.99 | 0.91 | 75 |
| *LMNA* | Syndrome | 0.70 | 0.34 | 14 |
| *LMNB2* | Syndrome | 0.77 | 0.48 | 19 |
| *LMX1A* | Candidate | 0.97 | 0.80 | 36 |
| *MADD* | GWAS | 0.99 | 0.89 | 54 |
| *MAFA* | Candidate | 0.28 | 0.07 | 5 |
| *MAFB* | Candidate | 0.79 | 0.47 | 19 |
| *MNX1/HLXB9* | Candidate | 0.39 | 0.12 | 6 |
| *MTNR1B* | GWAS | 0.87 | 0.72 | 34 |
| *MYT1* | Candidate | 0.92 | 0.72 | 33 |
| *NEUROD1* | MODY | 1.00 | 0.98 | 48 |
| *NEUROG3/NGN3* | NDM | 0.77 | 0.49 | 20 |
| *NKX2-2* | Candidate | 0.89 | 0.57 | 25 |
| *NKX6-1* | Candidate | 0.58 | 0.50 | 20 |
| *NOTCH2* | GWAS | 0.98 | 0.90 | 60 |
| *ONECUT1/HNF6* | Candidate | 0.78 | 0.53 | 22 |
| *PAX4* | MODY | 0.84 | 0.49 | 20 |
| *PAX6* | Candidate | 0.90 | 0.70 | 39 |
| *PBX1* | Candidate | 0.85 | 0.74 | 39 |
| *PDX1/IPF1* | MODY | 0.45 | 0.26 | 6 |
| *ZAC* | Syndrome | 0.94 | 0.66 | 31 |
| *PPARG* | GWAS, Syndrome | 1.00 | 0.94 | 61 |
| *PRC1* | GWAS | 0.96 | 0.89 | 73 |
| *PROX1* | GWAS | 1.00 | 0.98 | 61 |
| *PTF1A* | Candidate | 0.44 | 0.36 | 5 |
| *PTPRD* | GWAS | 0.98 | 0.92 | 80 |
| *RBMS1* | GWAS | 1.00 | 0.94 | 60 |
| *RFX6* | NDM | 0.94 | 0.86 | 72 |
| *SLC16A1* | CHI | 1.00 | 0.98 | 61 |
| *SLC2A2* | GWAS | 1.00 | 0.96 | 66 |
| *SLC30A8* | GWAS | 1.00 | 0.93 | 96 |
| *SOX2* | Candidate | 0.77 | 0.54 | 25 |
| *SOX4* | Candidate | 0.59 | 0.27 | 11 |
| *SOX9* | Candidate | 0.81 | 0.39 | 17 |
| *SREBF1* | Candidate | 0.47 | 0.17 | 8 |
| *SRR* | GWAS | 0.97 | 0.87 | 50 |
| *SYT9* | Candidate | 0.88 | 0.84 | 56 |
| *TCF7L2* | GWAS | 0.92 | 0.70 | 34 |
| *THADA* | GWAS | Not present on the capture array | | |
| *TMEM195* | GWAS | 0.97 | 0.92 | 62 |
| *TP53INP1* | GWAS | 1.00 | 0.97 | 84 |
| *TSPAN8* | GWAS | 0.98 | 0.87 | 54 |
| *UCP2* | Candidate | 0.98 | 0.75 | 39 |
| *VEGFA* | GWAS | 0.74 | 0.66 | 37 |
| *VPS13C* | GWAS | 0.98 | 0.89 | 57 |
| *WFS1* | GWAS, Syndrome | 0.82 | 0.64 | 34 |
| *ZBED3* | GWAS | 0.29 | 0.23 | 2 |
| *ZFAND6* | GWAS | 0.99 | 0.93 | 60 |

Abbreviations:

CHI: Gene known to cause Congenital Hyperinsulinism of Infancy

GWAS: Locus identified in Genome-Wide Association Studies of diabetes-related traits according to Grarup et al. (2010), Curr. Diab. Rep. 10:485-497.

Candidate: Gene suggested as Candidate for monogenic diabetes of unknown etiology by Edghill et al. (2010). J. Pancreas 11: 14-17 and from Oliver-Krasinski JM, Stoffers DA (2008). Genes Dev 22: 1998-2021.

MODY: Gene known to cause Maturity-Onset Diabetes of the Young

NDM: Gene known to cause Neonatal Diabetes Mellitus
