# Supplementary material for: Exome Sequencing and Genetic Testing for MODY
Source: PLoS One. 2012 May 25;7(5):e38050. doi: 10.1371/journal.pone.0038050 (PMC3360646; doi:10.1371/journal.pone.0038050)
Supplement: Table S2 — Fraction of target bases covered at minimum 8 X for each sample and gene. (DOC) [file pone.0038050.s003.doc]

**Table S2**

Fraction of target bases covered at minimum 8X for each sample and gene

| Genes | P01 | P02 | P03 | P04 | P05 | P06 | P07 | P08 | P09 | Mean  coverage |
| --- | --- | --- | --- | --- | --- | --- | --- | --- | --- | --- |
| *ABCC8* | 0.83 | 0.86 | 0.88 | 0.90 | 0.89 | 0.86 | 0.91 | 0.85 | 0.83 | 0.87 |
| *ADAM30* | 1.00 | 1.00 | 1.00 | 1.00 | 1.00 | 1.00 | 1.00 | 1.00 | 1.00 | 1.00 |
| *ADAMTS9* | 0.97 | 0.97 | 0.97 | 0.98 | 0.98 | 0.97 | 0.98 | 0.97 | 0.98 | 0.97 |
| *ADCY5* | 0.79 | 0.85 | 0.91 | 0.88 | 0.92 | 0.81 | 0.89 | 0.86 | 0.80 | 0.86 |
| *ADRA2A* | 0.63 | 0.63 | 0.58 | 0.66 | 0.62 | 0.58 | 0.66 | 0.60 | 0.61 | 0.62 |
| *AGPAT2* | 0.56 | 0.63 | 0.80 | 0.71 | 0.81 | 0.61 | 0.76 | 0.82 | 0.74 | 0.72 |
| *AKT2* | 0.86 | 0.84 | 0.92 | 0.93 | 0.95 | 0.90 | 0.96 | 0.93 | 0.83 | 0.90 |
| *ALMS1* | 0.97 | 0.98 | 0.98 | 0.97 | 0.98 | 0.98 | 0.98 | 0.98 | 0.97 | 0.98 |
| *APPL1* | 0.93 | 0.93 | 0.95 | 0.93 | 0.93 | 0.91 | 0.93 | 0.89 | 0.91 | 0.92 |
| *ARAP1* | 0.57 | 0.62 | 0.70 | 0.75 | 0.75 | 0.59 | 0.77 | 0.67 | 0.67 | 0.68 |
| *BCL11A* | 0.95 | 0.83 | 0.96 | 0.96 | 0.97 | 0.88 | 0.97 | 0.95 | 0.90 | 0.93 |
| *BLK* | 0.89 | 0.85 | 0.93 | 0.95 | 0.95 | 0.87 | 0.91 | 0.84 | 0.88 | 0.90 |
| *BSCL2* | 0.96 | 0.94 | 0.94 | 0.97 | 0.97 | 0.93 | 0.98 | 0.97 | 0.94 | 0.96 |
| *C2CD4B* | 0.00 | 0.08 | 0.10 | 0.15 | 0.00 | 0.00 | 0.05 | 0.10 | 0.00 | 0.05 |
| *CAMK1D* | 0.96 | 0.97 | 0.98 | 0.98 | 0.97 | 0.96 | 0.96 | 0.96 | 0.95 | 0.97 |
| *CAV1* | 0.94 | 0.98 | 1.00 | 0.98 | 0.97 | 0.89 | 1.00 | 1.00 | 0.97 | 0.97 |
| *CDC123* | 1.00 | 1.00 | 1.00 | 1.00 | 1.00 | 1.00 | 1.00 | 1.00 | 1.00 | 1.00 |
| *CDKAL1* | 0.99 | 1.00 | 1.00 | 1.00 | 1.00 | 1.00 | 1.00 | 0.99 | 1.00 | 1.00 |
| *CDKN2A* | 0.72 | 0.71 | 0.82 | 0.83 | 0.74 | 0.70 | 0.81 | 0.77 | 0.71 | 0.76 |
| *CDKN2B* | 0.72 | 0.73 | 0.92 | 0.91 | 0.89 | 0.74 | 0.82 | 0.73 | 0.58 | 0.78 |
| *CEL* | 0.65 | 0.67 | 0.70 | 0.75 | 0.79 | 0.65 | 0.81 | 0.69 | 0.75 | 0.72 |
| *CHCHD9* | 0.88 | 1.00 | 0.97 | 1.00 | 0.99 | 0.99 | 1.00 | 0.93 | 1.00 | 0.97 |
| *CISD2/WFS2* | 0.94 | 0.99 | 1.00 | 1.00 | 1.00 | 0.94 | 1.00 | 1.00 | 1.00 | 0.99 |
| *CRY2* | 0.85 | 0.86 | 0.90 | 0.92 | 0.93 | 0.85 | 0.90 | 0.84 | 0.85 | 0.88 |
| *DUSP8* | 0.41 | 0.41 | 0.43 | 0.52 | 0.44 | 0.36 | 0.44 | 0.48 | 0.35 | 0.43 |
| *DUSP9* | 0.60 | 0.49 | 0.63 | 0.63 | 0.63 | 0.64 | 0.60 | 0.58 | 0.45 | 0.58 |
| *EIF2AK3* | 0.99 | 1.00 | 1.00 | 1.00 | 1.00 | 0.97 | 1.00 | 1.00 | 0.99 | 0.99 |
| *FADS1* | 0.87 | 0.82 | 0.86 | 0.82 | 0.88 | 0.80 | 0.86 | 0.81 | 0.80 | 0.84 |
| *FOXA1* | 0.81 | 0.81 | 0.79 | 0.88 | 0.82 | 0.81 | 0.84 | 0.74 | 0.76 | 0.81 |
| *FOXA2* | 0.61 | 0.62 | 0.61 | 0.70 | 0.67 | 0.57 | 0.66 | 0.64 | 0.66 | 0.64 |
| *FOXA3* | 0.69 | 0.72 | 0.73 | 0.79 | 0.72 | 0.73 | 0.80 | 0.65 | 0.62 | 0.72 |
| *FTO* | 0.92 | 0.95 | 0.94 | 0.95 | 0.94 | 0.93 | 0.94 | 0.95 | 0.94 | 0.94 |
| *FXN* | 0.75 | 0.75 | 0.75 | 0.75 | 0.75 | 0.75 | 0.75 | 0.75 | 0.75 | 0.75 |
| *G6PC2* | 0.97 | 0.99 | 1.00 | 1.00 | 1.00 | 0.99 | 1.00 | 0.97 | 0.99 | 0.99 |
| *GATA4* | 0.61 | 0.61 | 0.62 | 0.66 | 0.63 | 0.61 | 0.66 | 0.63 | 0.62 | 0.63 |
| *GATA6* | 0.58 | 0.49 | 0.64 | 0.69 | 0.68 | 0.51 | 0.67 | 0.58 | 0.53 | 0.60 |
| *GCK* | 0.72 | 0.81 | 0.87 | 0.86 | 0.83 | 0.79 | 0.86 | 0.87 | 0.82 | 0.83 |
| *GCKR* | 0.99 | 1.00 | 1.00 | 1.00 | 1.00 | 1.00 | 1.00 | 1.00 | 0.99 | 1.00 |
| *GIPR* | 0.60 | 0.63 | 0.75 | 0.75 | 0.68 | 0.56 | 0.69 | 0.62 | 0.63 | 0.66 |
| *GLIS3* | 0.93 | 0.91 | 0.93 | 0.92 | 0.92 | 0.87 | 0.93 | 0.89 | 0.90 | 0.91 |
| *GLUD1* | 0.93 | 0.95 | 0.97 | 0.96 | 0.94 | 0.93 | 0.93 | 0.93 | 0.93 | 0.94 |
| *HADH* | 0.86 | 0.88 | 0.95 | 0.94 | 0.95 | 0.91 | 0.92 | 0.91 | 0.90 | 0.91 |
| *HFE* | 0.97 | 0.98 | 1.00 | 1.00 | 1.00 | 0.96 | 1.00 | 0.97 | 0.98 | 0.98 |
| *HHEX* | 0.63 | 0.63 | 0.66 | 0.56 | 0.63 | 0.56 | 0.65 | 0.55 | 0.57 | 0.60 |
| *HMGA2* | 0.75 | 0.75 | 0.75 | 0.75 | 0.75 | 0.75 | 0.75 | 0.75 | 0.75 | 0.75 |
| *HNF1A* | 0.66 | 0.68 | 0.72 | 0.77 | 0.79 | 0.63 | 0.83 | 0.70 | 0.66 | 0.72 |
| *HNF1B* | 0.86 | 0.81 | 0.88 | 0.91 | 0.89 | 0.85 | 0.88 | 0.90 | 0.86 | 0.87 |
| *HNF4A* | 0.82 | 0.85 | 0.97 | 0.87 | 0.94 | 0.85 | 0.92 | 0.90 | 0.87 | 0.89 |
| *IDE* | 0.96 | 0.97 | 0.97 | 0.97 | 0.97 | 0.96 | 0.97 | 0.95 | 0.95 | 0.96 |
| *IGF1* | 0.98 | 1.00 | 1.00 | 1.00 | 1.00 | 1.00 | 1.00 | 0.95 | 1.00 | 0.99 |
| *IGF2BP2* | 0.87 | 0.92 | 0.94 | 0.96 | 0.93 | 0.86 | 0.95 | 0.88 | 0.93 | 0.92 |
| *INS* | 0.52 | 0.55 | 0.69 | 0.64 | 0.62 | 0.48 | 0.68 | 0.51 | 0.56 | 0.58 |
| *INS-IGF2* | 0.42 | 0.46 | 0.54 | 0.53 | 0.50 | 0.42 | 0.59 | 0.44 | 0.46 | 0.48 |
| *INSM1* | 0.42 | 0.35 | 0.62 | 0.50 | 0.44 | 0.29 | 0.52 | 0.36 | 0.41 | 0.43 |
| *INSR* | 0.89 | 0.86 | 0.93 | 0.92 | 0.94 | 0.91 | 0.94 | 0.92 | 0.88 | 0.91 |
| *IRS1* | 0.86 | 0.82 | 0.90 | 0.89 | 0.88 | 0.78 | 0.91 | 0.83 | 0.83 | 0.86 |
| *ISL1* | 0.98 | 0.96 | 0.94 | 0.98 | 0.97 | 0.94 | 0.99 | 0.95 | 0.94 | 0.96 |
| *ITGB6* | 0.98 | 0.99 | 0.99 | 0.99 | 1.00 | 0.98 | 1.00 | 1.00 | 0.98 | 0.99 |
| *JAZF1* | 0.93 | 0.92 | 0.93 | 0.92 | 0.94 | 0.79 | 0.94 | 0.88 | 0.90 | 0.91 |
| *KCNJ11* | 0.99 | 0.99 | 1.00 | 0.99 | 0.97 | 1.00 | 0.99 | 1.00 | 1.00 | 0.99 |
| *KCNQ1* | 0.66 | 0.66 | 0.74 | 0.77 | 0.76 | 0.67 | 0.76 | 0.67 | 0.66 | 0.71 |
| *KLF11* | 0.93 | 0.88 | 0.93 | 0.93 | 0.92 | 0.93 | 0.92 | 0.92 | 0.91 | 0.92 |
| *KLF14* | 0.24 | 0.32 | 0.35 | 0.39 | 0.35 | 0.21 | 0.40 | 0.34 | 0.43 | 0.34 |
| *LGR5* | 0.99 | 0.98 | 0.99 | 1.00 | 1.00 | 1.00 | 1.00 | 0.96 | 0.99 | 0.99 |
| *LMNA* | 0.64 | 0.62 | 0.75 | 0.75 | 0.72 | 0.65 | 0.73 | 0.75 | 0.70 | 0.70 |
| *LMNB2* | 0.69 | 0.82 | 0.84 | 0.83 | 0.80 | 0.71 | 0.82 | 0.75 | 0.70 | 0.77 |
| *LMX1A* | 0.95 | 0.97 | 0.98 | 0.99 | 1.00 | 0.90 | 1.00 | 0.97 | 0.95 | 0.97 |
| *MADD* | 0.97 | 0.99 | 0.98 | 0.99 | 0.99 | 0.99 | 1.00 | 0.99 | 0.98 | 0.99 |
| *MAFA* | 0.16 | 0.35 | 0.37 | 0.46 | 0.20 | 0.25 | 0.39 | 0.25 | 0.10 | 0.28 |
| *MAFB* | 0.84 | 0.71 | 0.85 | 0.82 | 0.83 | 0.71 | 0.81 | 0.78 | 0.78 | 0.79 |
| *MNX1/HLXB9* | 0.33 | 0.35 | 0.52 | 0.50 | 0.49 | 0.35 | 0.42 | 0.23 | 0.31 | 0.39 |
| *MTNR1B* | 0.85 | 0.83 | 0.85 | 0.93 | 0.91 | 0.85 | 0.87 | 0.90 | 0.82 | 0.87 |
| *MYT1* | 0.85 | 0.93 | 0.90 | 0.93 | 0.93 | 0.93 | 0.95 | 0.93 | 0.91 | 0.92 |
| *NEUROD1* | 1.00 | 1.00 | 1.00 | 1.00 | 1.00 | 1.00 | 1.00 | 1.00 | 1.00 | 1.00 |
| *NEUROG3* | 0.71 | 0.80 | 0.90 | 0.81 | 0.78 | 0.68 | 0.73 | 0.65 | 0.84 | 0.77 |
| *NKX2-2* | 0.84 | 0.81 | 0.89 | 0.87 | 0.90 | 0.94 | 0.99 | 0.82 | 0.91 | 0.89 |
| *NKX6-1* | 0.56 | 0.53 | 0.66 | 0.62 | 0.56 | 0.53 | 0.72 | 0.54 | 0.52 | 0.58 |
| *NOTCH2* | 0.97 | 0.97 | 0.98 | 0.98 | 0.99 | 0.98 | 0.99 | 0.97 | 0.98 | 0.98 |
| *ONECUT1/HNF6* | 0.72 | 0.80 | 0.80 | 0.89 | 0.81 | 0.73 | 0.83 | 0.67 | 0.79 | 0.78 |
| *PAX4* | 0.73 | 0.78 | 0.92 | 0.88 | 0.84 | 0.85 | 0.89 | 0.81 | 0.88 | 0.84 |
| *PAX6* | 0.86 | 0.83 | 0.91 | 0.95 | 0.93 | 0.89 | 0.96 | 0.89 | 0.88 | 0.90 |
| *PBX1* | 0.84 | 0.85 | 0.86 | 0.84 | 0.84 | 0.84 | 0.86 | 0.84 | 0.85 | 0.85 |
| *PDX1* | 0.40 | 0.45 | 0.48 | 0.46 | 0.48 | 0.43 | 0.48 | 0.43 | 0.40 | 0.45 |
| *PLAGL1/ZAC* | 0.94 | 0.91 | 0.96 | 1.00 | 0.95 | 0.88 | 0.99 | 0.93 | 0.86 | 0.94 |
| *PPARG* | 1.00 | 1.00 | 1.00 | 1.00 | 1.00 | 0.99 | 1.00 | 1.00 | 0.99 | 1.00 |
| *PRC1* | 0.95 | 0.95 | 0.98 | 0.99 | 0.96 | 0.95 | 0.96 | 0.95 | 0.95 | 0.96 |
| *PROX1* | 1.00 | 1.00 | 1.00 | 1.00 | 1.00 | 1.00 | 1.00 | 1.00 | 1.00 | 1.00 |
| *PTF1A* | 0.42 | 0.46 | 0.46 | 0.44 | 0.45 | 0.44 | 0.46 | 0.44 | 0.43 | 0.44 |
| *PTPRD* | 0.97 | 0.97 | 0.98 | 0.98 | 0.99 | 0.98 | 0.99 | 0.97 | 0.98 | 0.98 |
| *RBMS1* | 1.00 | 1.00 | 1.00 | 1.00 | 1.00 | 0.99 | 1.00 | 0.99 | 1.00 | 1.00 |
| *RFX6* | 0.93 | 0.90 | 0.97 | 0.96 | 0.98 | 0.93 | 0.96 | 0.91 | 0.92 | 0.94 |
| *SLC16A1* | 1.00 | 1.00 | 1.00 | 1.00 | 1.00 | 1.00 | 1.00 | 1.00 | 1.00 | 1.00 |
| *SLC2A2* | 0.99 | 1.00 | 1.00 | 1.00 | 1.00 | 1.00 | 1.00 | 1.00 | 1.00 | 1.00 |
| *SLC30A8* | 0.99 | 1.00 | 1.00 | 1.00 | 0.99 | 1.00 | 1.00 | 1.00 | 0.99 | 1.00 |
| *SOX2* | 0.78 | 0.80 | 0.89 | 0.76 | 0.70 | 0.72 | 0.85 | 0.78 | 0.68 | 0.77 |
| *SOX4* | 0.59 | 0.61 | 0.64 | 0.63 | 0.61 | 0.40 | 0.62 | 0.60 | 0.61 | 0.59 |
| *SOX9* | 0.78 | 0.76 | 0.84 | 0.86 | 0.77 | 0.68 | 0.87 | 0.83 | 0.88 | 0.81 |
| *SREBF1* | 0.41 | 0.40 | 0.52 | 0.55 | 0.57 | 0.35 | 0.56 | 0.40 | 0.45 | 0.47 |
| *SRR* | 0.95 | 0.95 | 0.99 | 0.97 | 0.96 | 0.97 | 0.98 | 0.96 | 1.00 | 0.97 |
| *SYT9* | 0.88 | 0.88 | 0.88 | 0.88 | 0.89 | 0.88 | 0.88 | 0.88 | 0.88 | 0.88 |
| *TCF7L2* | 0.92 | 0.92 | 0.95 | 0.94 | 0.94 | 0.92 | 0.94 | 0.85 | 0.89 | 0.92 |
| *TMEM195* | 0.94 | 0.97 | 0.99 | 0.99 | 0.99 | 0.95 | 1.00 | 0.94 | 0.96 | 0.97 |
| *TP53INP1* | 1.00 | 1.00 | 1.00 | 1.00 | 1.00 | 1.00 | 1.00 | 0.98 | 0.99 | 1.00 |
| *TSPAN8* | 0.97 | 0.96 | 1.00 | 1.00 | 1.00 | 0.94 | 0.99 | 0.96 | 0.98 | 0.98 |
| *UCP2* | 0.99 | 0.90 | 1.00 | 1.00 | 0.98 | 0.98 | 0.97 | 0.99 | 1.00 | 0.98 |
| *VEGFA* | 0.74 | 0.74 | 0.73 | 0.75 | 0.75 | 0.72 | 0.76 | 0.74 | 0.73 | 0.74 |
| *VPS13C* | 0.98 | 0.98 | 0.99 | 1.00 | 1.00 | 0.97 | 1.00 | 0.95 | 0.96 | 0.98 |
| *WFS1* | 0.77 | 0.81 | 0.82 | 0.84 | 0.85 | 0.81 | 0.88 | 0.82 | 0.81 | 0.82 |
| *ZBED3* | 0.27 | 0.26 | 0.34 | 0.26 | 0.28 | 0.26 | 0.34 | 0.32 | 0.26 | 0.29 |
| *ZFAND6* | 0.96 | 1.00 | 0.99 | 1.00 | 1.00 | 1.00 | 1.00 | 1.00 | 0.97 | 0.99 |
| Candidate  gene set | 0.85 | 0.86 | 0.89 | 0.90 | 0.89 | 0.85 | 0.90 | 0.86 | 0.86 | 0.87 |
