# Supplementary material for: Exome Sequencing and Genetic Testing for MODY
Source: PLoS One. 2012 May 25;7(5):e38050. doi: 10.1371/journal.pone.0038050 (PMC3360646; doi:10.1371/journal.pone.0038050)
Supplement: Table S3 — Comparison between heterozygous genotypes obtained from the Affymetrix 6.0 chip and exome sequencing. (DOC) [file pone.0038050.s004.doc]

**Table S3**

Comparison between heterozygous genotypes obtained from the Affymetrix 6.0 chip and exome sequencing. All heterozygous SNPs obtained from 7800 good quality SNPs on the Affymetrix 6.0 chip present in the exome capture region.

| ID | No. of heteroz SNPs in array data | Called in exome data | Fraction called | Concordant |
| --- | --- | --- | --- | --- |
| P01 | NA | NA | NA | NA |
| P02 | 2228 | 1998 | 89.7% | 99.9% |
| P03 | 2310 | 2090 | 90.5% | 100.0% |
| P04 | 2267 | 2091 | 92.2% | 100.0% |
| P05 | 2219 | 2034 | 91.7% | 100.0% |
| P06 | NA | NA | NA | NA |
| P07 | 2199 | 2029 | 92.3% | 100.0% |
| P08 | 2142 | 1916 | 89.4% | 99.9% |
| P09 | 2229 | 1977 | 88.7% | 99.9% |

NA: Not Applicable (samples were not genotyped by Affymetrix 6.0 chip).
